# Surface-structure search with variable composition and periodicity via machine learning and evolutionary algorithms: applications to Pt/Ge oxidation and Au–Sn alloying

**DOI:** 10.1080/14686996.2026.2688059

**Published:** 2026-07-07

**Authors:** F. Kuroda, M. Otani

**Affiliations:** a Materials DX Research Center, National Institute of Advanced Industrial Science and Technology (AIST), Tsukuba, Japan; b Center for Computational Sciences (CCS), University of Tsukuba, Tsukuba, Japan

**Keywords:** Sections, lists, figures, tables, mathematics, fonts, references, appendices

## Abstract

First-principles structure prediction is essential for discovering functional materials; however, surface structure searches remain challenging because most search algorithms assume fixed in-plane periodicity and composition. Here we develop a global search framework that treats both two-dimensional superlattice periodicity and stoichiometry as dynamic variables, enabling the direct identification of the most stable surface structures across competing supercell shapes and compositions. The proposed method integrates an evolutionary algorithm with surface-specific variation operators and symmetry-enriched initialization, and accelerates screening via Bayesian optimization using atomic cluster expansion descriptors. Case studies on FCC Pt(111) and diamond Ge(100) surfaces yield oxygen-induced surface structures consistent with experimental observations, and the same framework identifies Sn alloying motifs on FCC Au(111) that agree with reported surface-structure trends. Overall, the framework delivers accurate structure prediction with substantially fewer high-cost first-principles evaluations and provides a general route to exploring complex materials landscapes – such as heterogeneous catalysis, electronics, and spintronics – in which coupled structural and compositional degrees of freedom govern functionality.

## IMPACT STATEMENT

This study presents an efficient first-principles framework for predicting complex surface structures without assuming fixed surface periodicity or composition. By combining evolutionary search, symmetry-informed initialization, and Bayesian optimization with atomic cluster expansion descriptors, the method reduces costly first-principles calculations while accurately identifying stable surface structures. Its successful applications to oxidized Pt(111), oxidized Ge(100), and Sn-alloyed Au(111) demonstrate its potential for accelerating materials discovery in catalysis, electronics, and spintronics.

## GRAPHICAL ABSTRACT

**Figure:**
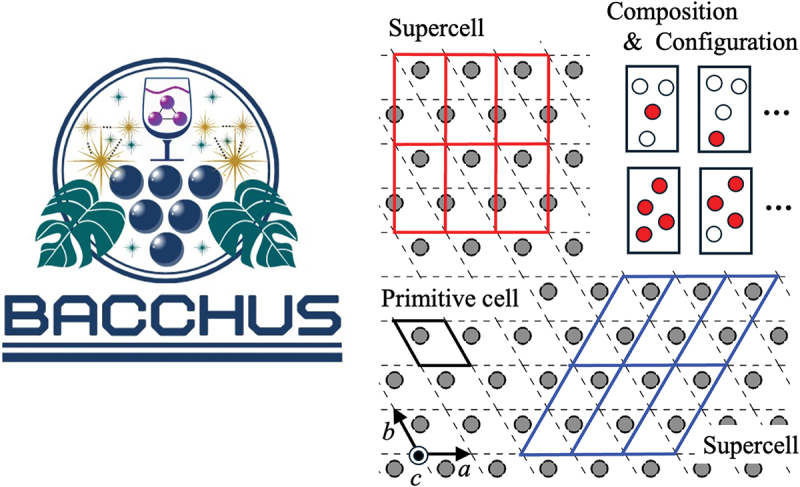

## 1. Introduction

Atomic structure is the most fundamental information in crystalline materials, determining their properties and functionalities. Once a structural topology is specified, a precise structural model and a wide range of physical properties can be computed using modern quantum-mechanical methods. In particular, the atomic structure of surfaces and interfaces plays a central role in functional materials, governing key processes in heterogeneous catalysis [1–3] and enabling emergent phenomena in spintronics [4,5]. However, identifying the atomic structures realized experimentally is often challenging, especially for surfaces and interfaces, where structural motifs can be diverse and complex.

**Figure 1.**
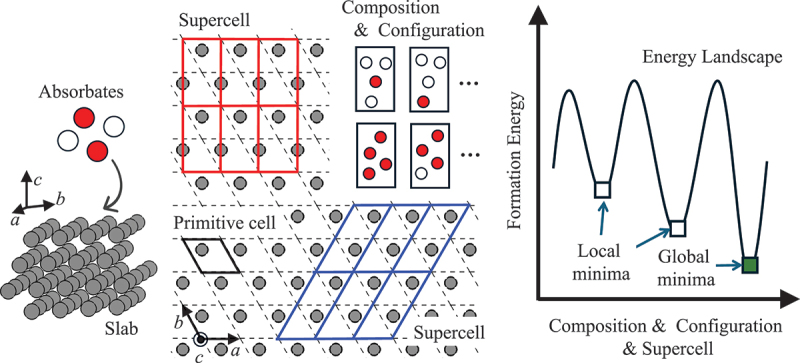
Schematic illustration of surface-structure search with variable periodicity and composition.

This difficulty stems from the vastness of the configurational space [6]: even for relatively small systems, the number of distinct local minima increases rapidly with the number of atoms, leading to a rugged energy landscape populated by numerous metastable structures. As a result, exhaustive enumeration is intractable, and local relaxation can easily become trapped far from the global minimum. The problem is further complicated in practical surface searches (Figure 1), where the in-plane periodicity and stoichiometry are frequently fixed a priori. While such constraints simplify the search, they can introduce a strong bias and may preclude the true ground state when the most stable structure occurs at a different superlattice periodicity, coverage, or composition. Consequently, a reliable framework should explore not only atomic coordinates but also the degrees of freedom associated with periodicity and stoichiometry, enabling unbiased competition among candidate reconstructions.

Crystal structure prediction (CSP) aims to identify low-energy atomic configurations on a highly rugged potential-energy surface [7,8]. To explore such complex landscapes efficiently, many CSP frameworks adopt population-based global search strategies for structure generation. Among them, particle-swarm optimization (PSO) updates a population of ‘particles’ using collective search dynamics; CALYPSO (Crystal structure AnaLYsis by Particle Swarm Optimization) [9–11] is a CSP package explicitly built on PSO, whereas evolutionary algorithms (EA) generate new candidates through variation operators such as crossover, mutation. A representative EA-based framework is USPEX (Universal Structure Predictor: Evolutionary Xtallography) [6,12–15], which is characterized by its rich set of variation EA operators for generating new candidate structures. Furthermore, programs that combine EA with Bayesian optimization (BO) include CrySPY (Crystal Structure prediction in Python) [16,17] and GOFEE (Global Optimization with First-principles Energy Expressions) [18,19]. These frameworks accelerate the search for low-energy structures by generating candidate configurations via EA and then selectively prioritizing expensive evaluations (such as first-principles calculations) using BO guided by a surrogate model, thereby reducing the total number of energy/relaxation calculations required.

USPEX, CALYPSO, and GOFEE can also be applied to slab models, enabling CSP on surfaces. In typical workflows, however, the degrees of freedom treated as variables differ across codes. GOFEE and CALYPSO usually explore structures within a fixed simulation cell and fixed stoichiometry, optimizing mainly the atomic coordinates in the prescribed slab supercell. In contrast, USPEX can perform variable-composition searches for slab/surface problems [15], allowing the stoichiometry to change during the search, while the in-plane surface cell (periodicity) is generally kept fixed to the user-defined slab supercell.

Although user-defined slab supercells reduce computational cost, they require manual preparation of multiple candidate cells to examine different surface periodicities. Because the combinations of surface superlattices and compositions increase combinatorially, this fixed-cell treatment can substantially limit the accessible structure-search space. Further details are provided in Appendix A.

In this study, we propose a surface-structure prediction method that simultaneously explores atomic coordinates, surface superlattice periodicity, and chemical composition within a unified search space. The method couples an EA equipped with surface-specific variation operators and symmetry-enriched initialization to a Bayesian-optimization layer, thereby accelerating the discovery of low-energy reconstructions under less restrictive constraints.

The paper is outlined as follows. First, we describe the details of the proposed method in Sections 2.1–2.4, including random structure generation, parent selection, evolutionary operations, and candidate evaluation via BO. Then, we present three case studies to which the proposed framework is applied. In Sections 2.5, we briefly describe the structural relaxation procedure and the energy evaluation settings used for these three case studies. As the first of the three case studies, we examine oxidation on FCC Pt(111), which is of central importance for heterogeneous catalysis and electrocatalysis. In this case study, we also discuss whether the BO-driven CSP scheme provides an effective optimization strategy for identifying low-energy surface structures. In addition, we discuss surface reconstruction and oxidation on diamond Ge(100), a post-Si semiconductor, and finally examine Au-Sn alloys on the FCC Au(111) surface, which have attracted attention as catalytic materials and as Rashba-splitting systems.

## 2. Method

**Figure 2.**
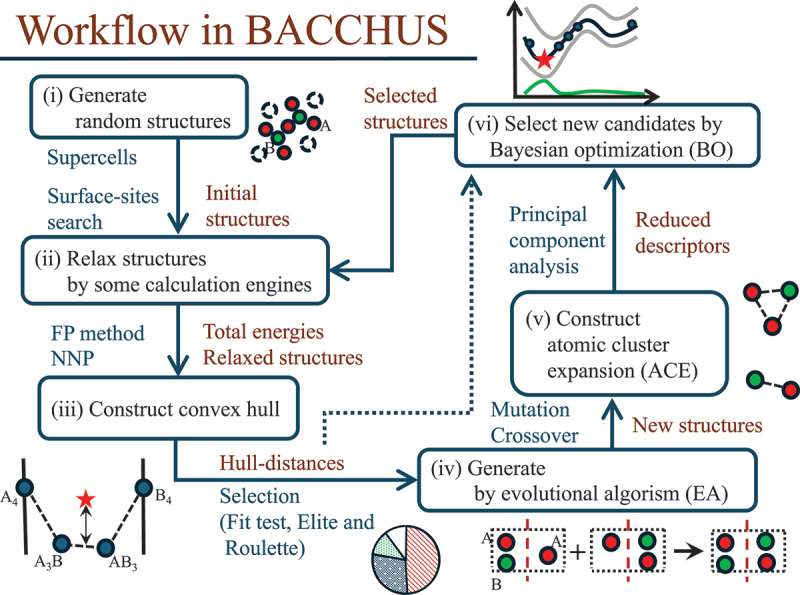
Schematic BACCHUS workflow.

First of this section provides an computational workflow (Figure 2) of the proposed surface-structure search framework, named BACCHUS (Bayesian Atomic Cluster Construction High-efficiently Unveiling of Surface-structures). In BACCHUS, (i) we first generate an initial pool of random structures by varying the supercell (in-plane periodicity) of slabs and adsorption configurations on slabs. (ii) Each structure is locally relaxed, and its total energy is evaluated using a chosen calculation engine, such as first-principles (FP) methods or neural network potentials (NNPs). (iii) From the resulting energies, we construct a convex hull and quantify the thermodynamic stability of each structure by its distance to the hull (energy above hull). (iv) Promising structures are then selected through a combination of fit-based screening, elite selection, and roulette-wheel selection, and are used as parents in an evolutionary algorithm. Offspring structures are generated via variation operators such as mutation and crossover, yielding on the order of $10^{3}$ new candidates per generation. (v)–(vi) Using the accumulated dataset, we next build an Gaussian-process regression model based on atomic cluster expansion (ACE) surrogate model and employ BO to prioritize the next candidates for exact energy evaluations. By iterating this loop from (ii) to (vi), BACCHUS efficiently explores the surface-structure space while substantially reducing the number of expensive FP calculations required.

Then, we describe each component of the workflow in detail from Sections 2.1–2.5.

### 2.1. Random structure generation

Initial structures are generated by random sampling. In BACCHUS, we treat both the in-plane supercell periodicity and the stoichiometry as dynamic variables. Therefore, we first generate random supercells by varying symmetrically distinct lattice vectors within user-defined supercell size (see Section 2.1.1). Next, we place adsorbates on the slab surface by identifying adsorption sites based on the symmetry of the chosen superlattice (see Section 2.1.2).

#### 2.1.1. Supercell construction

We generate derivative superlattices from a given parent slab structure using the method proposed by A. Santoro [20,21] and G. L. W. Hart [22]. Starting from a parent cell of arbitrary lattice type, the procedure first enumerates all symmetry-distinct derivative superlattices, which are then used to construct the corresponding derivative structures. Consider the transformation

(1) B=HA,

where $A=(a_{1},a_{2}{)}^{T}$ is a 2×2 matrix whose row vectors are the parent in-plane lattice vectors $a_{i},i=1,2$. H is a 2×2 integer matrix, and $B=(b_{1},b_{2}{)}^{T}$ is the matrix of the superlattice vectors $b_{i},i=1,2$. When the determinant of H is |H|=±1, H simplly rotates the parent cell. This transformation H can be represented by a Hermite normal form (HNF) matrix:

(2) $$H=\left(\begin{array}{cc} h_{11} & 0\\ h_{21} & h_{22} \end{array}\right),$$

where $h_{11},h_{22}>0$ and $0\leq h_{21}<h_{22}$. The |H| is given by $h_{11}h_{22}$, which corresponds to the supercell size $n_{S}$ (the number of parent cells contained in the supercell). We can systematically enumerate all possible HNF matrices for a given $n_{S}$ by finding all integer pairs $\left(h_{11},h_{22},h_{21}\right)$ that satisfy the above conditions.

Then, we further reduce the number of superlattices by considering the symmetry of the parent lattice. In BACCHUS, the in-plane point group symmetry of the parent slab is identified using the spglib library [23], and we eliminate rotational operations {R}. If one obtains two supercell matrices with the same $n_{S}$, $B_{1}=H_{1}A$ and $B_{2}=H_{2}A$. These two supercells are considered equivalent, if they satisfy the relation $C=({\overset{ˉ}{R}B}_{1}^{T}{)}^{T}B_{2}^{-1}$, where C is an unimodular matrix and $\overset{ˉ}{R}$ is a rotational operation of the cartesionian coordinates.

**Figure 3.**
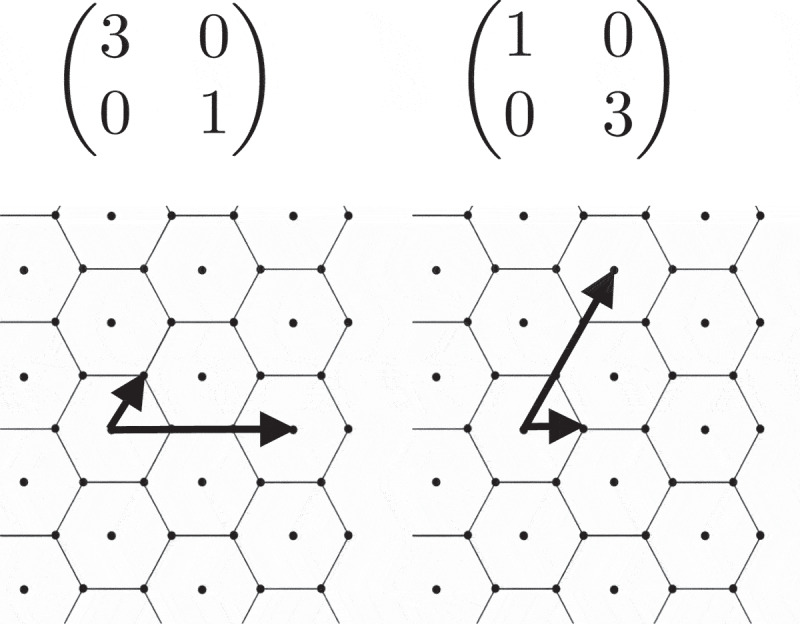
Two symmetry-equivalent supercells on FCC (111) surface.

For example (also see Appendix A), we consider the point group system of the FCC (111) surface. For $n_{S}=3$, we can generate four HNF matrices from the above procedure. However, two diagonal matrices in these four are found to be equivalent by the rotational symmetry (Figure 3).

#### 2.1.2. Symmetry-enriched initialization

**Figure 4.**
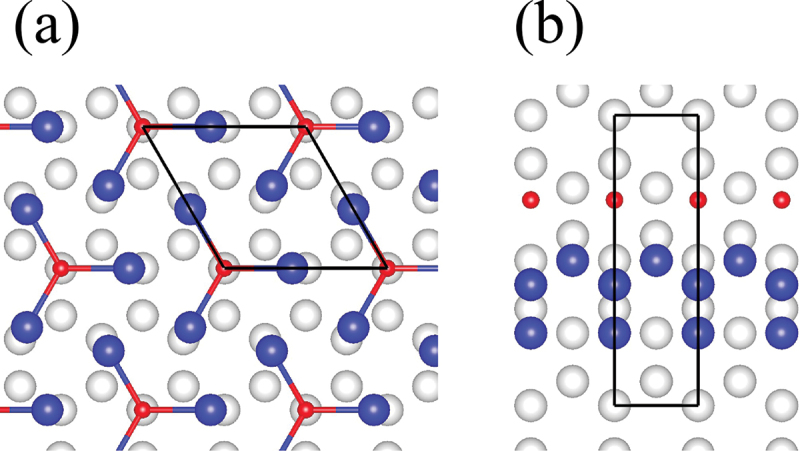
Symmetry-enriched site generation on a supercell surface. (a) three Fold rotational symmetry, (b) mirror symmetry.

Initial structures are generated by placing symmetric sites on the slab surface of each supercell. We fist identify point group symmetry of the supercell surface using following relation: $R_{S}=H^{-1}\mathrm{RH}$, where $R_{S}$ should be an integer matrix if R is a symmetry operation of the given supercell. From this relation, we can construct the point symmetry group of the supercell surface. Moreover, the symmetry subgroup of the supercell surface can be obtained. Next, we discretize the supercell surface with a uniform two-dimensional mesh and compile a list of candidate positions. Using the symmetry group (including a selected subgroup) obtained above, we classify these mesh points into symmetry-equivalent sets. We then choose point sets that remain invariant under the symmetry operations and place adsorbates on them to generate symmetry-preserving initial configurations. This symmetry-enriched initialization allows us to sample adsorption structures that respect the supercell surface symmetry by construction. Figure 4 shows examples of symmetry-enriched site generation on a supercell surface.

### 2.2. Parent structures selection

**Figure 5.**
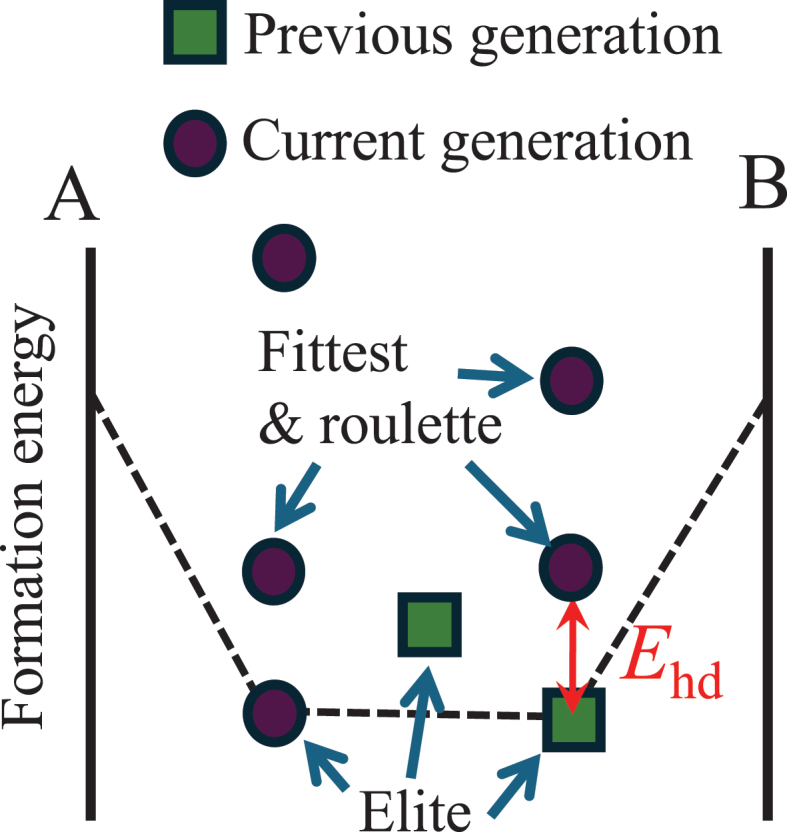
Schematic view of the formation-energy convex hull and the parent-selection procedure.

After evaluating the energies of all structures in the current generation, we select parent structures for the evolutionary operations. We first construct the convex hull of formation energies from the evaluated structures. The formation energy $E_{\mathrm{form}}$ of an adsorbate structure containing $N_{A}$ atoms of element A and $N_{B}$ atoms of element B is defined as

(3) $$E_{\mathrm{form}}=E_{\mathrm{slab}+\mathrm{ads}}-n_{S}E_{\mathrm{slab}}-N_{A}\mu_{A}-N_{B}\mu_{B},$$

where $E_{\mathrm{slab}+\mathrm{ads}}$ is the total energy of the slab with adsorbates, $E_{\mathrm{slab}}$ is the energy per parent cell of the clean slab, $n_{S}$ is the supercell size (i.e. the number of parent cells in the supercell), and $\mu_{A}$ ($\mu_{B}$) is the chemical potential of adsorbates of element A (B). Unless otherwise noted, $\mu_{A}$ and $\mu_{B}$ are set to the energies per atom in their most stable phases. From the convex hull, we compute the energy above hull, referred to as the hull distance $E_{\mathrm{hd}}$, for each structure as a measure of its thermodynamic stability (Figure 5) [24]. After computing $E_{\mathrm{hd}}$ for all structures, we select parent structures using a combination of three schemes: elite, fittest, and roulette. In elite selection, we select the structures with the lowest formation energies. This scheme retains the most stable structures across all generations. In contrast, fittest and roulette selection are applied to structures in the current generation, where $N_{\mathrm{st}}$ structures are available after local relaxation and energy evaluation.

In fittest selection, we introduce two threshold parameters, $E_{hd}^{min}$ and $r_{\mathrm{fit}}$. Here, $E_{hd}^{min}$ is a minimum hull-distance threshold used to exclude structures that are too similar to the candidates already retained by elite selection. $r_{\mathrm{fit}}(0<r_{\mathrm{fit}}<1)$ is the fittest ratio: we sort the $N_{\mathrm{st}}$ structures in ascending order of $E_{\mathrm{hd}}$ and select the lowest-$E_{\mathrm{hd}}$ top $N_{\mathrm{st}}r_{\mathrm{fit}}$ structures.

In roulette selection, $N_{\mathrm{rou}}$ structures are selected from $N_{\mathrm{st}}r_{\mathrm{fit}}$ structures probabilistically based on their $E_{\mathrm{hd}}$ values. The selection probability $p_{i}$ of structure i is defined as

(4) $$p_{i}=\frac{{f{\;}^{\prime }}_{i}}{\sum_{i}f_{i}{\;}^{\prime }},$$

(5) $$f{{\;}^{\prime }}_{i}=\frac{a-b}{f_{\max}-f_{\min}}\;f_{i}+\frac{bf_{\max}-af_{\min}}{f_{\max}-f_{\min}},$$

(6) $$f_{i}=-1*E_{\mathrm{hd}}(i),$$

where $f_{\max}$ and $f_{\min}$ are the maximum and minimum values of $f_{i}$ in the population, and a and b are user-defined parameters.

### 2.3. Offspring structure generation

**Figure 6.**
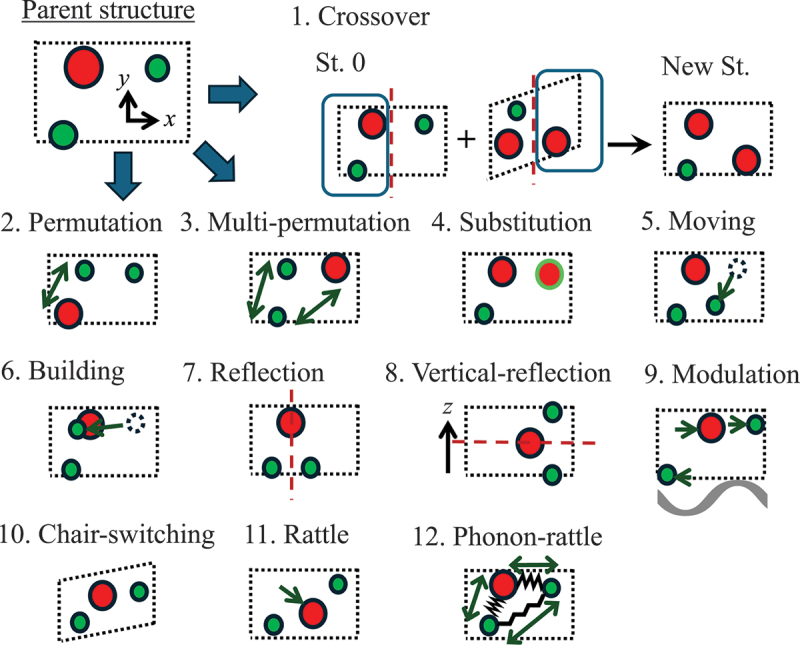
Evolutionary operations implemented in BACCHUS. Different colors represent different element types.

The purpose of the evolutionary operations is to generate more global structural diversity while retaining locally stable atomic motifs inherited from low-energy parents. Using the parent structures selected in Section 2.2, we generate offspring structures via evolutionary operations. In BACCHUS, we implement *crossover and eleven mutation operators* (Figure 6)*: single-permutation, multi-permutation, substitution, moving, building, reflection, vertical-reflection, modulation, chair-switching, rattle, and phonon-rattle*.

For crossover, two parent structures are each split approximately in half, and the resulting fragments are combined (stitched together) to create offspring structures. For single-permutation and multi-permutation, we randomly select one or more adsorbates and change their element types to other species. In substitution, we randomly select an adsorbate and replace it with a different adsorbate species. In moving, we randomly select an adsorbate and significantly change its lateral position while keeping its z coordinate fixed. In building, we randomly select adsorbates and stack them on top of another adsorbate to create a cluster-like structure. In reflection, we reflect the positions of adsorbates with respect to a vertical plane passing through a surface atom. In vertical-reflection, we reflect the adsorbate positions with respect to a horizontal plane passing through a surface atom. In modulation, we apply a sinusoidal modulation to the adsorbate positions along a chosen direction. In chair-switching, we switch the adsorbate configuration by using a different supercell lattice. In rattle, we randomly displace the adsorbates by a distance within a prescribed range. In phonon-rattle, phonon modes are first obtained for a given structure using the finite-displacement method. The adsorbates are then displaced along the phonon eigenvectors with amplitudes determined from the corresponding eigenvalues, following the procedure proposed by West [25] and implemented in the hiPhive package [26].

In addition, we assign an element-specific cutoff radius to each species. During structure generation, if any pair of atoms approaches closer than the corresponding cutoff distance (defined from the element-specific radii), we perform a short structural relaxation using a simple classical repulsive potential with a characteristic length on the order of the cutoff, thereby removing unphysical atomic overlaps and stabilizing the generated structures.

### 2.4. Offspring structure evaluation

After generating offspring structures via evolutionary operations, we select candidates for expensive energy evaluations using BO. In BACCHUS, we construct ACE-based descriptors for BO and use them to evaluate the auction function for all candidates. Finally, we select the top $N_{\mathrm{eval}}$ candidates with the lowest auction function values for exact energy evaluations. In the following, we briefly describe ACE-based descriptors and the BO method used in BACCHUS.

#### 2.4.1. Atomic cluster expansion (ACE)

To build an accurate surrogate model, it is crucial to encode local atomic environments around each atom with high fidelity. Various descriptors for atomic environments have been proposed, including the Oganov – Valle fingerprint [27], the smooth overlap of atomic positions (SOAP) [28], Behler – Parrinello symmetry functions [29], and moment tensor potentials (MTP) [30]. In CrySPY, the Oganov – Valle fingerprint is used, whereas GOFEE employs an Oganov – Valle-type descriptor augmented with angular terms. Among these approaches, the atomic cluster expansion (ACE) [31] provides a systematic framework for constructing a (in principle) complete basis for representing local atomic environments. In BACCHUS, we use ACE descriptors generated with ACEpotentials.jl [32].

ACE descriptors are constructed as follows. We define atom-centered basis coefficients by projecting one-particle basis functions onto the neighbor density around atom i:

(7) $$A_{i,\mathrm{znlm}}=\sum_{j\in N_{i}}\psi_{\mathrm{znlm}}(r_{\mathrm{ij}},Z_{i},Z_{j}),$$

where $r_{\mathrm{ij}}$ is the relative position vector from atom i to atom j, and $N_{i}$ denotes the set of neighbors within a cutoff radius. Here, z and z denote the elemental species. The one-particle basis functions ψ are given by

(8) $$\psi_{\mathrm{znlm}}(r_{\mathrm{ij}},Z_{i},Z_{j})=R_{\mathrm{nl}}(r_{\mathrm{ij}})\;Y_{\mathrm{lm}}({\hat{r}}_{\mathrm{ij}})\;\delta_{zZ_{j}},$$

where $r_{\mathrm{ij}}=∥r_{\mathrm{ij}}∥$ and ${\hat{r}}_{\mathrm{ij}}=r_{\mathrm{ij}}/r_{\mathrm{ij}}$, $R_{\mathrm{nl}}(r)$ is a radial basis function (with a cutoff), and $Y_{\mathrm{lm}}(\hat{r})$ is a spherical harmonic. The explicit form of $R_{\mathrm{nl}}(r)$ is taken from the implementation in ACEpotentials.jl, and this implementation assume $R_{\mathrm{nl}}(r)=R_{n}(r)$, i.e. the radial function is independent of l.

Using these one-particle coefficients, product basis functions up to v-body order are constructed as

(9) $${\tilde{A}}_{i,\mathrm{znlm}}=\prod_{t=1}^{v}A_{i,z_{t}n_{t}l_{t}m_{t}},$$

where $(z,n,l,m)=(z_{t},n_{t},l_{t},m_{t}{)}_{t=1}^{v}$ denotes a multi-index. From these products, rotationally invariant ACE features are obtained as

(10) $$B_{i}=C\;{\tilde{A}}_{i},$$

where ${\tilde{A}}_{i}$ is the vector whose components are ${\tilde{A}}_{i,\mathrm{znlm}}$, and C is a sparse linear operator that performs the contraction (equivalently, integration over the rotation group O(3)) to ensure rotational invariance. In practice, the matrix elements of C can be expressed in terms of Wigner 3j symbols. The resulting vector $B_{i}$ constitutes the ACE descriptor for atom i and encodes v-body geometric correlations in a symmetry-consistent manner.

#### 2.4.2. Dimensionality reduction and descriptors

**Figure 7.**
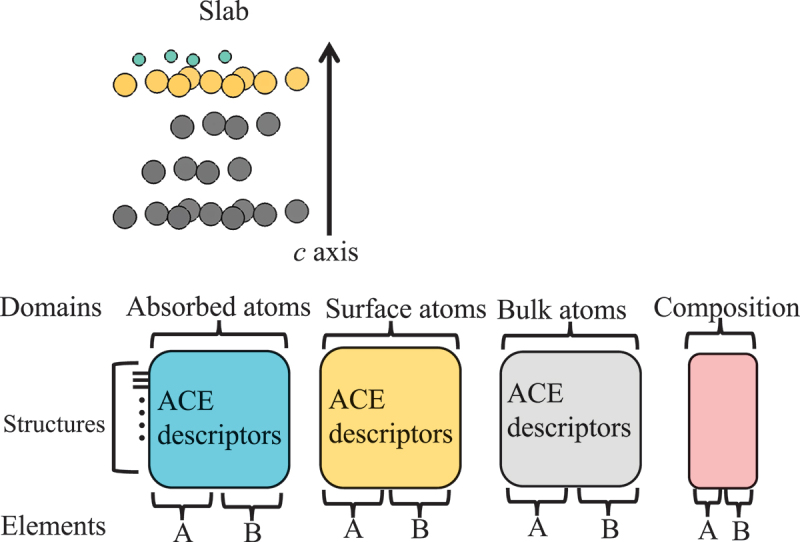
Schematic illustration of the ACE descriptor matrices. Light blue, yellow, and gray spheres represent adsorbate, surface, and bulk atoms, respectively.

The ACE descriptor $B_{i}$ for each atom i in a structure is computed as described in Section 2.4.1. However, the dimensionality of $B_{i}$ can be quite high, which increases the computational cost of GPR. To mitigate this issue, we first average the ACE descriptors over atoms of the same element type (z) within each predefined domain (Figure 7). Here, a domain refers to one of the following groups of atoms: (i) adsorbate atoms, (ii) surface (substrate) atoms, or (iii) bulk atoms. Moreover, we add the composition ratios of each element type within the adsorbate to the descriptor vector. Then, we apply principal component analysis (PCA), which is implemented in the Scikit-learn package [33], to reduce the dimensionality of the resulting descriptor vector. The length of the final descriptor vector can be determined by specifying the desired cumulative contribution ratio of PCA.

#### 2.4.3. Bayesian optimization (BO)

In BACCHUS, we employ BO using PYSBO library [34]. The PYSBO library provides a random feature map-based GPR implementation, which is enabled to avoid the computationally expensive training process. This method can be described as bellow.

Given a training set $D=\{(x_{i},y_{i}){\}}_{i=1}^{M}$, where $x_{i}$ denotes the descriptor of structure i (e.g. an ACE-based representation) and $y_{i}$ is its hull distance, GPR assumes that the training targets $y=(y_{1},\ldots ,y_{M}{)}^{T}$ follow a multivariate Gaussian distribution,

(11) p(y|D)=N(μ,Σ).

When the Bayesian linear regression model is used, y can be expressed as

(12) $$y(x)=w^{T}\phi (x)+\varepsilon ,$$

where *w* is the coefficient vector, ϕ(x) is the feature vector of x, and ε is noise generated according to a Gaussian distribution N(0,σ). Let $\Phi \in R^{M\times \ell }$ be the design matrix whose i-th row is $\phi (x_{i}{)}^{T}$, and let $y\in R^{M}$ be the vector of training targets. Then, under the Bayesian linear regression model with Gaussian noise variance $\sigma^{2}$, the mean vector μ and covariance matrix Σ of the joint Gaussian p(y|D)=N(μ,Σ) are given by

(13) $$\mu ={\left(\Phi^{T}\Phi +\sigma^{2}I\right)}^{-1}\Phi y,$$

(14) $$\Sigma =\sigma^{2}{\left(\Phi^{T}\Phi +\sigma^{2}I\right)}^{-1},$$

where I is the identity matrix and $\Phi =(\phi (x_{1}),\ldots ,\phi (x_{M}))$. From this method, the Gaussian kernel $k(x,x^{\prime })$ can be approximated as

$$k(x,x^{\prime })=\exp\left(-\frac{||x-x{\prime ||}^{2}}{2\eta^{2}}\right)$$

(15) $$≃\phi (x{)}^{T}\phi (x^{\prime }),$$

(16) $$\phi (x)=(z_{w_{1},\beta_{1}}(x/\eta ),\ldots ,z_{w_{\ell },\beta_{\ell }}(x/\eta ){)}^{T},$$

where $z_{w,\beta }(x)=\sqrt{2}\cos(w^{T}x+\beta )$, and d-dimensional random vectors $w_{i}$ is generated from the probability $2\pi^{-d/2}\exp(-||w|{|}^{2}/2)$. β is uniformly sampled from [0,2π). Using the above GPR model, BO selects the next M+1-th data vaule $x_{M+1}$ by the aquistion function. In this study, we use expected improvement (EI) as the acquisition function. The EI expected value (E) of how much $y_{\max}$ updates when x is observed:

$$\mathrm{EI}(x)=E[\max(0,y_{\mathrm{best}}-y(x))],$$

(17) $$=\sigma_{c}\left(x\right)[t(x)F(t\left(x)\right)+f(t\left(x)\right)],$$

where $t\left(x\right)=(\mu_{c}\left(x\right)-y_{\mathrm{best}})/\sigma_{c}$. Here, f is the probability density of N(0,1), and $\mu_{c}$ and $\sigma_{c}$ can be computed from the mean and standard deviation of the posterior distribution:

p(y|D)=p(w|D)

(18) $$=N(\mu =\frac{1}{\sigma^{2}}A^{-1}\Phi y,\Sigma =A^{-1}).$$

Here, the matrix A is defined as $A=\Phi \Phi^{T}/\sigma^{2}+I$.

### 2.5. Exact energy evaluation

Following the procedures described in Section 2.4, we obtain candidate structures that require high-fidelity energy evaluations. In principle, these exact evaluations are performed using FP calculations based on density functional theory (DFT). However, although BO optimizes the number of structures that should be evaluated, FP calculations remain computationally expensive when applied to a large number of candidates. To reduce the number of costly FP evaluations, we employ a universal neural network potential (UNN) to pre-relax and screen structures. In this work, we use the pretrained MACE [35] foundation potential medium-mpa-0 for this purpose. By combining UNN-based pre-evaluation with selective FP refinement, we substantially lower the overall computational cost while retaining FP-level accuracy for the final reported energetics.

## 3. Results

In this section, we present three case studies to which the proposed BACCHUS framework is applied. The parameters used for the BACCHUS framework in each case study are summarized in Appendix B. The details of the computational settings for structural relaxation and energy evaluation are provided in Appendix C. Moreover, in Appendix D, we validate the accuracy of the MACE potential used for pre-evaluation by comparing its predictions with FP. First, we examine oxidation on FCC Pt(111) surface in Section 3.1 and discuss the effectiveness of the BO-driven CSP scheme. Next, in Section 3.2, we investigate surface reconstruction and oxidation on diamond Ge(100) surface. Finally, in Section 3.3, we explore Au – Sn alloys on FCC Au(111) surface.

### 3.1. Pt – O surface structures on FCC Pt(111)

We first apply the proposed BACCHUS framework to the oxidation of the FCC Pt(111) surface, which is of central importance for heterogeneous catalysis and electrocatalysis. To validate the effectiveness of the BO-driven CSP scheme, we also perform a reference EA-based search without BO acceleration, in which candidates for exact energy evaluations are selected randomly.

For this benchmark, we use a supercell size of $n_{S}=16$ and a total coverage of θ=1.0 monolayer (ML), while varying the oxygen composition within the range of 0.25–0.90. In other words, we search surface structures spanning compositions from Pt${\;}_{12}$O${\;}_{4}$ to Pt${\;}_{2}$O${\;}_{14}$. At each EA generation, the algorithm produces approximately 3000 offspring structures, from which we select 55 structures for exact (high-fidelity) energy evaluations. Each structural search is repeated three times starting from different randomly generated initial populations.

**Figure 8.**
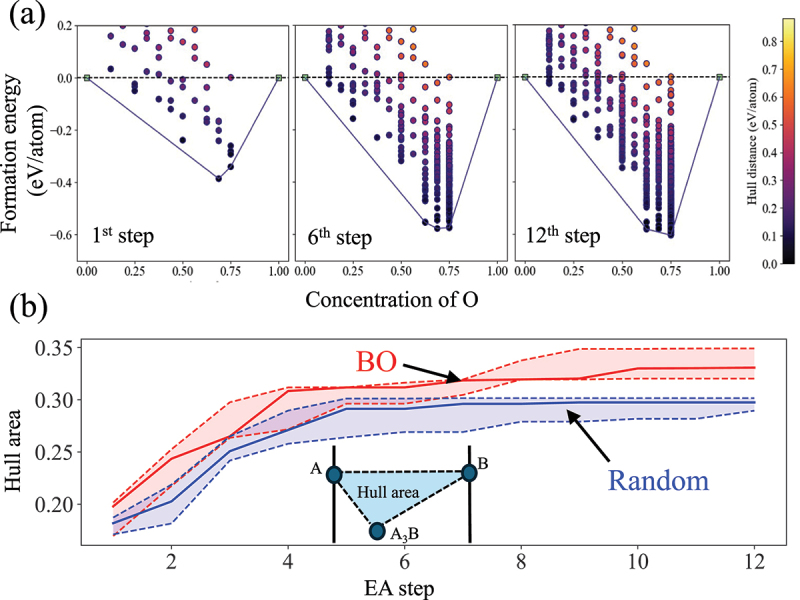
(a) Evolution of the formation-energy convex hull obtained by the BO-driven EA search. (b) Comparison of the growth of the convex-hull area between the EA search without BO (random selection) and the BO-driven search. Solid lines indicate the median, while dashed lines represent the minimum and maximum values.

Figure 8(a) shows the evolution of the formation-energy convex hull obtained by the BO-driven EA search. Here, the convex-hull area is defined as the area enclosed by the convex hull and the two end-member points (FCC Pt and O${\;}_{2}$). The BO-driven EA search progressively discovers lower-energy structures across compositions compared with the initial random pool through iterative generations, and the convex hull obtained after the 12th generation exhibits a substantially deeper hull than the initial one. Figure 8(b) compares the growth of the convex-hull area between the EA search without BO (random selection) and the BO-driven search. From this figure, the BO-driven search increases the convex-hull area more rapidly than the random-selection baseline, indicating that incorporating BO based on ACE descriptors (as mentioned in Section 2.4) can substantially accelerate the EA-based search.

From Figure 8(a), we further confirm the efficiency of the BO-driven search and observe that Pt – O structures with higher oxygen concentrations tend to be more stable. Motivated by this trend, we next focus on searching stable structures in an oxygen-rich regime (composition range of 0.50–0.75). Here, we set $n_{S}=8$, which is smaller than in the benchmark above. To compensate for the smaller supercell, we increase the total coverage from θ=1.0 to θ=1.5. That is, we span compositions from Pt${\;}_{6}$O${\;}_{6}$ to Pt${\;}_{3}$O${\;}_{9}$. Under these conditions, we search for low-energy surface structures and compare them with structures reported in experimental studies.

**Figure 9.**
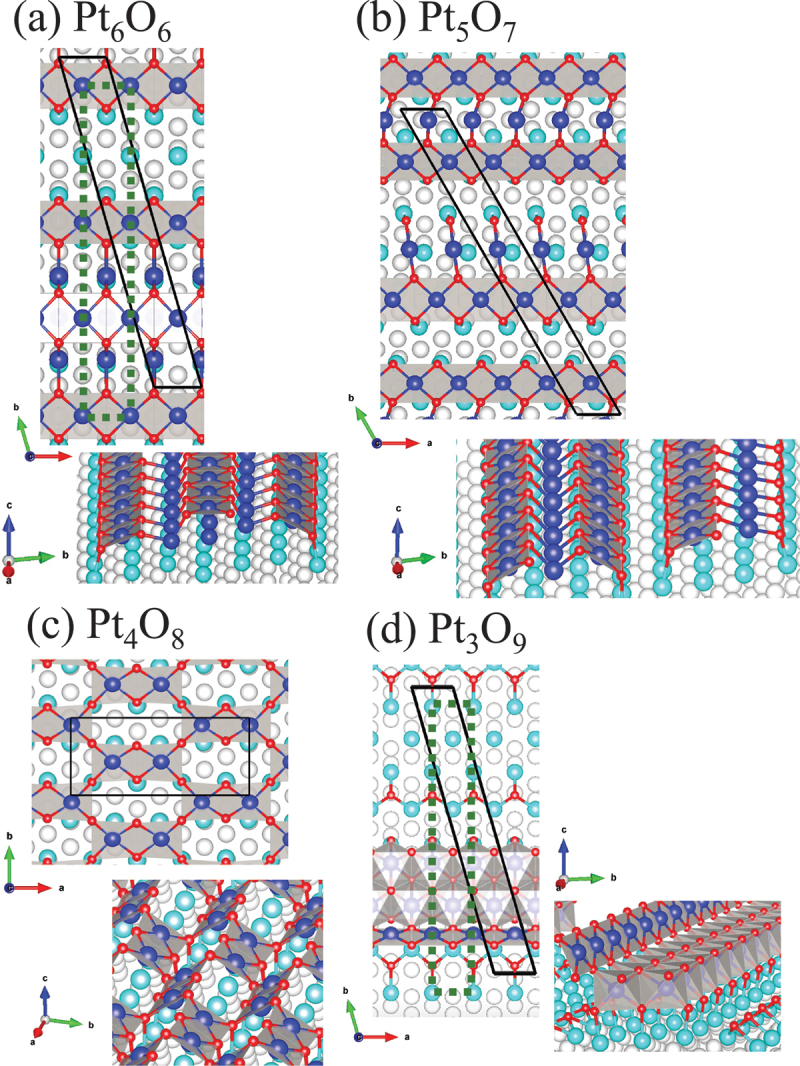
The lowest-energy structure at each composition for Pt oxidation on FCC Pt (111). Blue spheres indicate Pt adatoms on the surface, light-blue spheres indicate the topmost Pt layer in the slab, white spheres indicate the remaining Pt atoms in the slab, and red spheres indicate O atoms.

**Figure D1.**
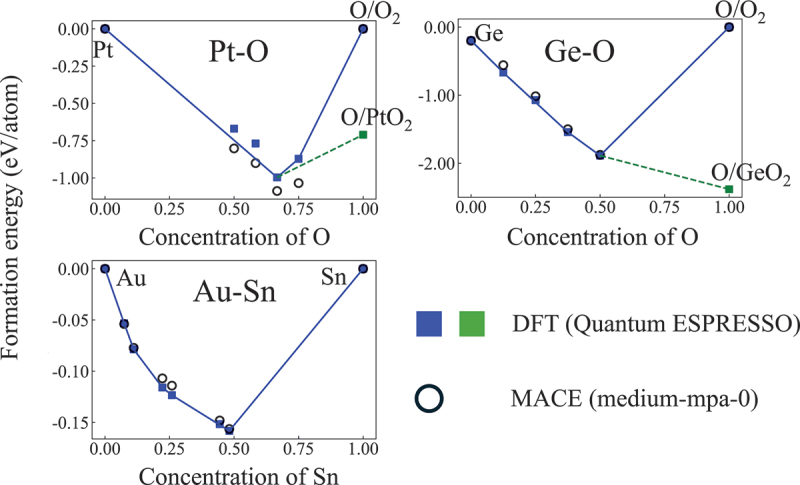
Formation energy comparison with DFT (QE) and MACE (medium-mpa-0).

Figures 9(a–d) show the lowest-energy structure at each composition obtained using the BACCHUS framework. For Pt${\;}_{6}$O${\;}_{6}$, Pt${\;}_{5}$O${\;}_{7}$, and Pt${\;}_{4}$O${\;}_{8}$, the most stable configurations feature square-planar PtO${\;}_{4}$ units, in which four oxygen atoms are coordinated around a Pt atom, and neighboring units share edges. Similar square-planar PtO${\;}_{4}$ motifs also appear in bulk Pt${\;}_{3}$O${\;}_{4}$ [36]. In particular, the PtO${\;}_{4}$ units in the Pt${\;}_{6}$O${\;}_{6}$ and Pt${\;}_{5}$O${\;}_{7}$ surfaces exhibit a row-ordered arrangement on top of the underlying Pt adatoms, whereas those in Pt${\;}_{4}$O${\;}_{8}$ form a checkerboard-like pattern. At higher oxygen contents, the structure evolves into an edge-sharing network of PtO${\;}_{6}$ octahedra, as found for Pt${\;}_{3}$O${\;}_{9}$. Although the nearest-neighbor and second nearest-neighbor Pt – Pt distances in our PtO${\;}_{6}$-octahedral motifs are about 2.8 and 3.2 Å, respectively, similar PtO${\;}_{6}$-octahedral motifs are also present in bulk α-PtO${\;}_{2}$ (CdI${\;}_{2}$-type structure), where the nearest-neighbor Pt – Pt distance is 3.16 Å [36]. Although Pt${\;}_{3}$O${\;}_{9}$ is slightly less stable than the structures on the convex hull when the bulk-oxide chemical-potential limit is considered (see Figure D1 and Appendix D), its hull distance is small. Therefore, Pt${\;}_{3}$O${\;}_{9}$ may still be regarded as a possible metastable structure in the early stage of oxide-film growth.

In experimental studies using X-ray photoelectron spectroscopy (XPS) and scanning tunneling microscopy (STM) [37–40], Pt(111) oxidation has been reported to yield various ordered surface-oxide motifs, including row-ordered and other commensurate superstructures, depending on the preparation conditions. The row-ordered arrangements of PtO${\;}_{4}$ units identified here provide atomistic models for such ordered phases. Moreover, the formation of α-PtO${\;}_{2}$-like trilayers has also been reported experimentally. Therefore, the structures obtained in our calculations appear to be broadly consistent with the experimentally reported motifs, suggesting that the proposed framework captures key structural features of Pt(111) oxidation.

### 3.2. Ge and Ge – O surface structures on diamond Ge(100)

We focus on the surface reconstruction of diamond Ge(100), including the effects of oxidation. In this study, we search structures spanning the composition range from Ge${\;}_{8}$ to Ge${\;}_{4}$O${\;}_{4}$ with supercell size $n_{S}=4$.

**Figure 10.**
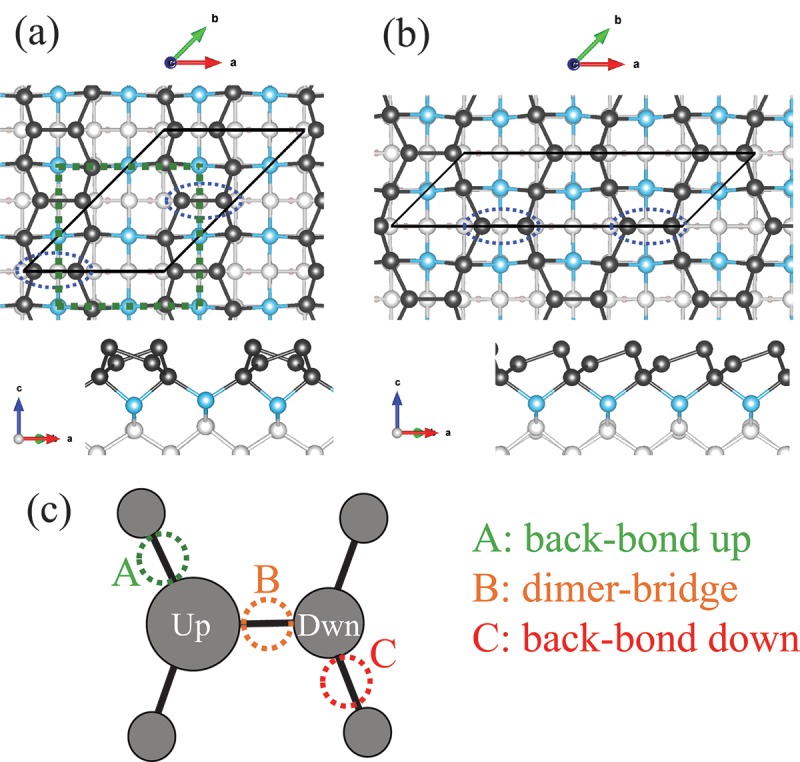
The lowest (a) and second-lowest (b) reconstruction structures of diamond Ge(100) obtained by this work. Black spheres indicate Ge adatoms on the surface, light-blue spheres indicate the topmost Ge layer in the slab, and white spheres indicate the remaining Ge atoms in the slab. Panel (c) schematically illustrates the adsorption site of an O atom on the widely discussed Ge dimer.

Figures 10(a,b) show the lowest and second-lowest-energy reconstruction structures of diamond Ge(100) obtained in this work. Both structures contain Ge dimers. The difference between them lies in the arrangement of the Ge dimers and in the buckling direction of the dimer atoms. The lowest-energy structure (a) exhibits anti-parallel dimer buckling. These degrees of freedom of the Ge dimers lead to the energy difference between the two reconstructions, which is estimated to be 110 meV per dimer from our DFT calculations. Previous DFT studies [41–44] have proposed two stable dimer arrangements, namely the c(4×2) and p(2×2) reconstructions, which are nearly degenerate in energy. The p(2×2) reconstruction corresponds to our lowest-energy structure. In experimental STM studies [44], both phases have been observed to coexist on the surface.

Then, we discuss the effect of oxidation on the Ge(100) surface reconstruction. Previous DFT studies [45–47] have proposed several adsorption configurations of an O atom on a Ge dimer (Figure 10(c)). In particular, the dimer-bridge site and the back-bond-down site have been reported to be energetically stable.

**Figure 11.**
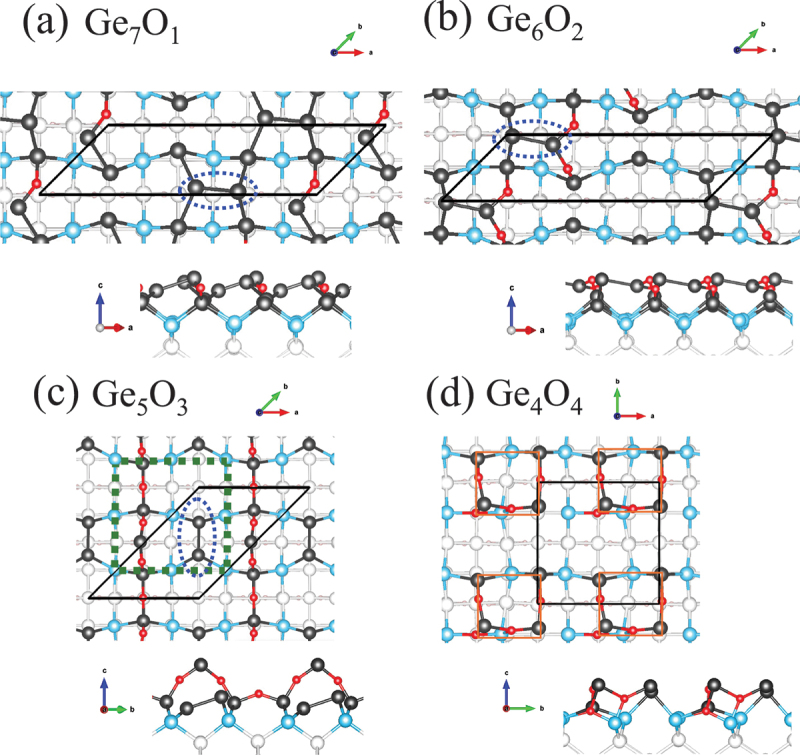
The lowest-energy structure at each composition for Ge oxidation on diamond Ge (100). Black spheres indicate Ge adatoms on the surface, light-blue spheres indicate the topmost Ge layer in the slab, white spheres indicate the remaining Ge atoms in the slab, and red spheres indicate O atoms.

In our study, the back-bond-down site is found to be preferred in the lowest-energy structures at each low-oxidation composition (Ge${\;}_{7}$O${\;}_{1}$ and Ge${\;}_{6}$O${\;}_{2}$ in Figure 11(a,b)). On the other hand, oxygen adsorption at the back-bond-down site induces a lateral shift of the Ge dimer rows. At higher oxidation levels (Ge${\;}_{5}$O${\;}_{3}$ and Ge${\;}_{4}$O${\;}_{4}$ in Figure 11(c,d)), more substantial structural changes occur. In Ge${\;}_{5}$O${\;}_{3}$, two O adatoms induce the protrusion of a Ge adatom. In Ge${\;}_{4}$O${\;}_{4}$, the Ge dimers vanish upon O adsorption, and the reconstructed Ge${\;}_{4}$O${\;}_{4}$ structure becomes condensed into a p(1×1) unit cell. In STM experiments [45,48–50], oxidation has been reported to progressively remove the Ge dimers. In addition, bright protrusions are observed in STM experiments, which have been theoretically attributed to Ge adatom, and a p(1×1) reconstruction emerges at higher oxidation levels. These experimental observations are consistent with our obtained structures: the disappearance of Ge dimers and the appearance of protruding Ge adatoms are captured in the Ge${\;}_{5}$O${\;}_{3}$ and Ge${\;}_{4}$O${\;}_{4}$ structures (Figure 11 (c,d)), and the condensation into a p(1×1) unit cell in Ge${\;}_{4}$O${\;}_{4}$ is consistent with the experimentally reported p(1×1) phase [45].

### 3.3. Au–Sn alloying on FCC Au (111)

**Figure 12.**
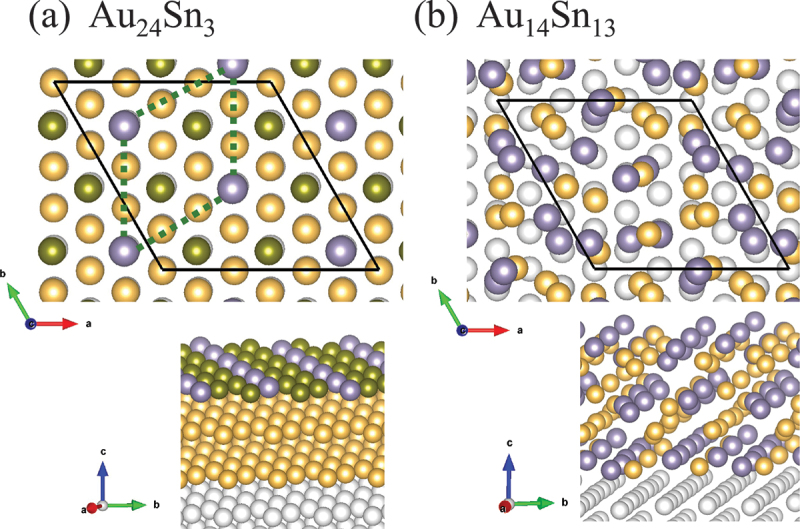
The lowest-energy structure at Au${\;}_{24}$Sn${\;}_{3}$ and Au${\;}_{14}$Sn${\;}_{13}$ on FCC Au (111). Dark gold spheres indicate topmost Au adatoms on the surface, gold spheres indicate the Au surface layer, white spheres indicate the remaining Au atoms in the slab, and silver spheres indicate Sn atoms.

To examine the performance of our framework for surface-alloy systems, we finally investigate Au – Sn alloying on FCC Au(111) using a supercell size of $n_{S}=9$ and a total coverage of θ=3 ML (Figure 12). At the Au${\;}_{24}$Sn${\;}_{3}$ composition, Sn atoms segregate to the surface and form a hexagonal overlayer; the resulting top-layer stoichiometry corresponds to Au${\;}_{2}$Sn. This Au${\;}_{2}$Sn surface-alloy motif is consistent with experimental STM studies [51–54] on Sn/Au(111), which report ordered Au${\;}_{2}$Sn surface-alloy phases and commensurate superstructures.

In addition, as a new insight obtained from the present theoretical approach, we found that, at the Sn-rich composition Au${\;}_{14}$Sn${\;}_{13}$, the system no longer preserves the underlying FCC-Au lattice; instead, the optimized structures exhibit a strongly disordered, “degraded” arrangement.

## 4. Conclusion

We have developed a global surface-structure search framework, BACCHUS, that simultaneously explores atomic coordinates, in-plane superlattice periodicity, and stoichiometry/coverage within a unified search space. By combining an evolutionary algorithm equipped with surface-specific variation operators and symmetry-enriched initialization with Bayesian optimization based on ACE descriptors, the method efficiently navigates highly rugged energy landscapes while reducing the number of expensive first-principles evaluations.

In the Pt(111) oxidation case study, the BO-driven search was shown to accelerate the growth of the formation-energy convex hull compared with a random-selection baseline, and it revealed oxygen-rich low-energy motifs consistent with experimentally discussed surface-oxide trends. For Ge(100), the framework reproduced the characteristic dimer reconstructions and captured oxidation-induced restructuring, including dimer-row shifts at low oxidation and the disappearance of dimers toward a p(1×1)-like phase at higher oxidation, in line with reported STM observations. Finally, for Au – Sn surface alloying on Au(111), the BACCHUS framework identified surface segregation and hexagonal ordering of Sn at Au${\;}_{24}$Sn${\;}_{3}$ with an Au${\;}_{2}$Sn-like top-layer stoichiometry consistent with experimental reports, while predicting strongly distorted, bulk-alloy-like arrangements at Sn-rich compositions such as Au${\;}_{14}$Sn${\;}_{13}$.

Because the present framework treats the surface periodicity as a search variable, it can, in principle, systematically explore long-period surface reconstructions. Nevertheless, complex long-period reconstructions, such as the Dimer – Adatom – Stacking-fault structure of the Si(111) 7 × 7 surface, remain challenging in practice because evolutionary structure searches generally become less efficient as the number of atoms and the system size increase [55].

The proposed framework provides a practical and general route to predicting complex surface and interface structures relevant to heterogeneous catalysis, electronics, and spintronics, where coupled structural and compositional degrees of freedom govern functionality. Future work will extend the approach to broader chemistries, environmental conditions, long-period reconstructions, and fully automated integration with high-throughput first-principles workflows.

## Figures and Tables

**Figure A1.**
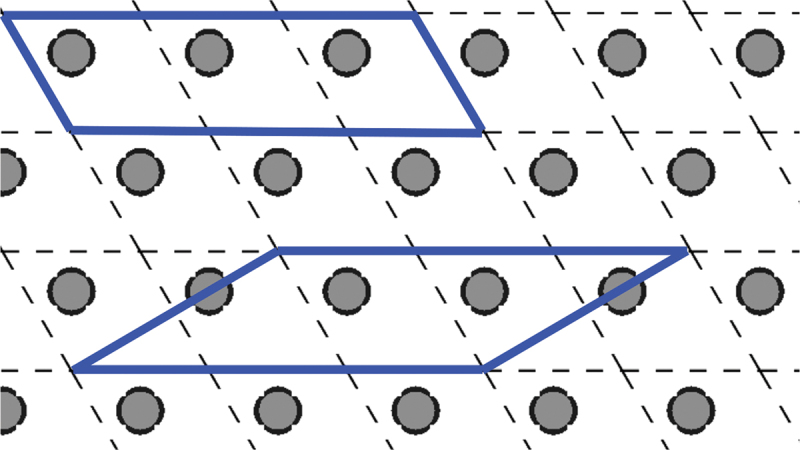
Schematic illustration of superlattice choices for a hexagonal lattice with $n_{s}=3$ Blue solid lines indicate the supercell lattices generated by the HNF representation with symmetry reduction.

## Impact statement

This study presents an efficient first-principles framework for predicting complex surface structures without assuming fixed surface periodicity or composition. By combining evolutionary search, symmetry-informed initialization, and Bayesian optimization with atomic cluster expansion descriptors, the method reduces costly first-principles calculations while accurately identifying stable surface structures. Its successful applications to oxidized Pt(111), oxidized Ge(100), and Sn-alloyed Au(111) demonstrate its potential for accelerating materials discovery in catalysis, electronics, and spintronics.

## Appendix A. Variation of surface superlattice choices

Here, we illustrate the restriction imposed by fixed user-defined slab supercells using the case of $n_{S}=3$ (Figure A1), where $n_{S}$ denotes the superlattice size, i.e. the area ratio of the surface supercell to the primitive surface unit cell. In the HNF representation (Eq. 2), four candidate supercells are generated for $n_{S}=3$ before symmetry reduction. After considering the symmetry of the hexagonal surface lattice, these four candidates are reduced to two symmetry-inequivalent supercell choices. For a larger superlattice size, the number of candidates increases further (the number of HNF candidates is given by the sum of the positive divisors of $n_{S}$); for example, 13 candidate supercells are generated for $n_{S}=9$ before symmetry reduction.

This example shows that many possible surface periodicities must be considered even for moderate superlattice sizes. In a fixed-cell approach, these candidate slab supercells have to be manually prepared and examined, and this burden increases further when different surface compositions are included. Therefore, fixed-cell treatments can substantially restrict the accessible structure-search space and may miss reconstructions requiring non-diagonal or differently shaped surface cells.

## Appendix B. Parameterization of the BACCHUS framework

In this appendix, we summarize the key parameters used in the BACCHUS framework for the case studies presented in the main text. In Eq. 5, the parameters were set to a=10 and b=1.

We assigned an element-specific cutoff radius to each species. The cutoff radii were set to 1.0 Å for Ge, 0.7 Å for O, 1.0 Å for Pt, 1.0 Å for Au, and 1.0 Å for Sn.

ACE descriptors were generated with a cutoff radius of 5.0 Å, and the maximum body order was set to 4.

For the reference chemical potentials used in the formation-energy calculations, $\mu_{A}$ and $\mu_{B}$ were evaluated as the total energy per atom of the corresponding reference phase calculated using the same computational settings. Specifically, diamond Ge, diamond Sn, an isolated O${\;}_{2}$ molecule, FCC Au, and FCC Pt were used as the reference phases for Ge, Sn, O, Au, and Pt, respectively. For oxygen, the reference chemical potential was taken as one half of the total energy of the isolated O${\;}_{2}$ molecule.

## Appendix C. Computational settings

In this appendix, we provide the computational settings used for structural relaxation and energy evaluation.

The global structure search and the associated energy/force evaluations were performed using the MACE pretrained model mace-mpa-0. During the search stage, structural relaxations were carried out only for the explored surface region (i.e. the adsorbates and the topmost surface layer), while the remaining slab atoms were kept fixed to reduce the computational cost.

After the search, the most stable structures for each composition were re-optimized using DFT as implemented in Quantum ESPRESSO (QE) [56,57]. In these QE calculations, all atoms including the slab layers were fully relaxed. We employed the Perdew – Burke – Ernzerhof (PBE) generalized gradient approximation (GGA) for the exchange – correlation functional [58]. Ultrasoft pseudopotentials (USPPs) [59] from the GBRV (Garrity – Bennett – Rabe – Vanderbilt) library [60] were used. For structural relaxations, the plane-wave cutoff and charge-density cutoff were set to 40 Ry and 400 Ry, respectively, with a 6×6×1 Monkhorst – Pack k-point mesh. For final total-energy evaluations, we used the higher cutoffs (50 Ry and 500 Ry) with a denser 12×12×1 k-point mesh.

To obtain reliable formation energies involving oxygen, we applied an overbinding correction to the DFT total energy of O${\;}_{2}$ within the GGA-PBE framework [61].

## Appendix D. Validation of MACE potential and discussion on thermodynamic stability

In this appendix, we validate the accuracy of the MACE potential used for pre-screening by comparing its predictions with DFT calculations. Figure D1 presents the comparison. For the Pt – O and Ge – O systems, we computed the formation energies of the lowest-energy structures at each composition. For the Au – Sn system, we computed the formation energies of the structures on the formation-energy convex hull. Based on this validation, we confirmed that the MACE potential provides sufficiently accurate relative energies for the pre-screening stage.

In addition, we estimate the oxygen chemical-potential limits from bulk oxide phases. Among the considered bulk oxide structures from the Materials Project database [62], PtO${\;}_{2}$ (hydrophilite-type structure) and GeO${\;}_{2}$ (rutile-type structure) are found to have the most negative formation energies for the Pt – O and Ge – O systems, respectively. The corresponding oxide-formation limits are evaluated as

(19) $$\mu_{O/PtO_{2}}=\frac{E_{PtO_{2}}^{b\mathrm{ulk}}-E_{Pt}^{bulk}}{2},$$

(20) $$\mu_{O/GeO_{2}}=\frac{E_{GeO_{2}}^{b\mathrm{ulk}}-E_{Ge}^{bulk}}{2},$$

where $E_{PtO_{2}}^{b\mathrm{ulk}}$ and $E_{GeO_{2}}^{b\mathrm{ulk}}$ are the total energies of bulk PtO${\;}_{2}$ and GeO${\;}_{2}$ of formula unit, respectively, and $E_{Pt}^{bulk}$ and $E_{Ge}^{bulk}$ are the total energies of bulk Pt and Ge, respectively. These chemical potentials represent the upper bounds of the oxygen chemical potential above which bulk oxide formation becomes thermodynamically favorable.

On the other hand, these oxide-derived chemical potentials correspond to the bulk-oxide limit, where the oxide layer grown on the substrate is sufficiently thick and can be regarded as a bulk crystalline phase. In the initial stage of oxide growth, the oxide layer is not fully developed, and surface, interface, and lattice-mismatch effects can contribute significantly to the total energy. Thus, the effective oxide energy may be written as $E_{o\mathrm{xide}}^{film}=E_{o\mathrm{xide}}^{bulk}+\Delta E^{surf/i\mathrm{nterface}}$. If the excess contribution $\Delta E^{surf/i\mathrm{nterface}}$ is positive, as is commonly expected for thin or incipient oxide layers, the corresponding oxide-formation chemical potential becomes higher than that estimated from the bulk oxide. Therefore, the chemical-potential limits derived from bulk PtO${\;}_{2}$ and GeO${\;}_{2}$ should be interpreted as bulk-oxide limits, while the actual formation threshold in the early stage of oxide growth may be shifted to higher oxygen chemical potentials.

In Figure D1, we also show the chemical-potential limits estimated from the corresponding bulk oxides. The results indicate that the oxide structures obtained by the present framework are stable with respect to these bulk-oxide limits, except for Pt${\;}_{3}$O${\;}_{9}$. Even for Pt${\;}_{3}$O${\;}_{9}$, however, the hull distance is small. Considering that surface, interface, and lattice-mismatch contributions can be significant in the initial stage of oxide-film growth, Pt${\;}_{3}$O${\;}_{9}$ may still exist as a metastable structure during the early oxidation process.
